# Cytotoxic Action of Carboxyborane Heterocyclic Amine Adducts

**DOI:** 10.1155/MBD.1997.229

**Published:** 1997

**Authors:** Merrill C. Miller, Anup Sood, Bernard F. Spielvogel, Ken Bastow, Iris H. Hall

**Affiliations:** 1 Division Medicinal Chemistry School of Pharmacy University of North Carolina Chapel Hill North Carolina 27559 USA; 2 Boron Biologicals, Inc. 620 Hutton St Suite 104 Raleigh North Carolina 27606 USA

## Abstract

The heterocyclic carboxyborane amines were found to be potent cytotoxic agents in the murine L1210 lymphoid leukemia and human HeLa suspended carcinoma cells. These agents were observed to inhibit HeLa DNA topoisomerase II activity ~ 200 μM and L1210 topoisomerase II activity ≥ 100 μM. These agents did not cause DNA protein linked breaks themselves, but upon incubation for 14-24 hr did enhance the ability of VP-16 to cause cleavable complexes. The heterocyclic amineboranes inhibited DNA synthesis and caused DNA strand scission. They were additive with VP-16 in affording these results as well as inhibiting colony growth of L1210 cells after co-incubation for 1 hr. The agents inhibited *in vitro* PKC phosphorylation of both L1210 lymphoid leukemia and human topoisomerase II enzyme.


### CYTOTOXIC ACTION OF CARBOXYBORANE HETEROCYCLIC AMINE ADDUCTS

Merrill .C. Miller, III[1], Anup Sood[2], Bernard F. Spielvogel[2], Ken Bastow[1] and Iris H. Hall*[1]

[1] Division Medicinal Chemistry, School of Pharmacy, University of North Carolina, Chapel Hill, North Carolina, 27559 USA
[2] Boron Biologicals, Inc. 620 Hutton St, Suite 104, Raleigh North Carolina, 27606, USA

### Abstract

The heterocyclic carboxyborane amines were found to be potent cytotoxic agents in the murine L1210 lymphoid leukemia and human HeLa suspended carcinoma cells. These agents were observed to inhibit HeLa DNA topoisomerase II activity ~ 200 μM and L1210 topoisomerase II activity $\geq$ 100 μM. These agents did not cause DNA protein linked breaks themselves, but upon incubation for 14-24 hr did enhance the ability of VP-16 to cause cleavable complexes. The heterocyclic amineboranes inhibited DNA synthesis and caused DNA strand scission. They were additive with VP-16 in affording these results as well as inhibiting colony growth of L1210 cells after co-incubation for 1 hr. The agents inhibited *in vitro* PKC phosphorylation of both L1210 lymphoid leukemia and human topoisomerase II enzyme.

### Introduction

Previously, a series of cyano-, carboxy-, carboxymethoxy-, and carbamoylborane adducts of heterocyclic amines were shown to be potent antineoplastic agents in the Lewis lung and P388 lymphocytic leukemia screens at 20 mg/kg/day in mice. Furthermore, they demonstrated significant cytotoxic activity in human $Tmolt_3$ lymphoblastic leukemia, HeLa-$S^3$ uterine carcinoma, KB nasopharynx, colorectal adenocarcinoma SW480, and osteosarcoma and murine L1210 lymphoid leukemia cells [1]. A mode of action study in L1210 cells demonstrated that DNA synthesis and purine syntheses were preferentially inhibited with significant inhibition at regulatory enzymatic sites such as PRPP-amido transferase, IMP dehydrogenase, dihydrofolate reductase, nucleoside kinases which resulted in a reduction of deoxyribonucleotide pools. Furthermore, L1210 DNA strand scission was evident after 24 hr. incubation with the agents, but the compounds did not alkylate the bases of DNA, intercalate between base pairs of DNA or cause cross linking of DNA strands [1]. Thus, the purpose of the following study is to characterize the reasons why the drugs cause DNA strand scission.

### Methods

Compounds **1-7** were previously synthesized and the chemical and physical characteristics reported [3] [Table 1]. All radioisotopes were purchased from New England Nuclear (Boston, MA) unless otherwise indicated. Radioactivity was determined in a Fisher Scintiverse scintillation fluid with correction for quenching. Substrates and cofactors were obtained from Sigma Chemical Co. (St. Louis, MO).

### Cytotoxicity

Compounds **1-7** were tested for cytotoxic activity by homogenizing drugs in a 1 mg/ml solution in 0.05% Tween 80/$H_2O$. These solutions were sterilized by passing them through an acrodisc (0.45 μ). The murine $L_{1210}$ lymphoid leukemia and the human HeLa-$S^3$ uterine carcinoma cell lines were maintained culture following standard literature techniques. Geran *et al.*'s protocol [2] was used to assess the cytotoxicity of the compounds and standards in each cell line. Values for cytotoxicity were expressed as $ED_{50}$ = μg/ml, i.e. the concentration of the compound inhibiting 50% of cell growth. $ED_{50}$ values were determined by the trypan blue exclusion technique. A value of less than 4 μg/ml was required for growth inhibition activity to be considered significant.

### Table 1 Structures of Compounds

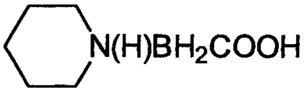

**1.** Piperidine carboxyborane

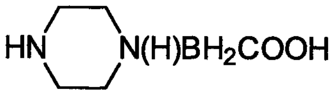

**2.** Piperazine carboxyborane

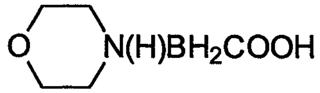

**3.** Morpholine carboxyborane

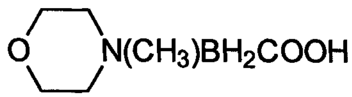

**4.** 4-Phenyl piperidine carboxyborane

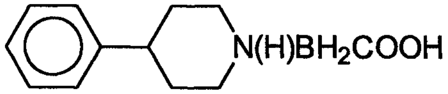

**5.** 4-Methyl piperidine carboxyborane

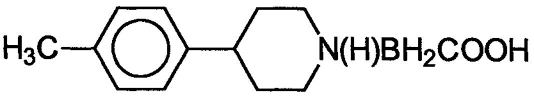

**6.** 4-Methyl piperidine carboxyborane

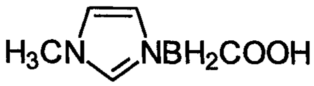

**7.** N-Methyl imidazole carboxyborane

### DNA Synthesis

Incorporation of $^3$H-thymidine into DNA of $10^6$ L1210 or HeLa-$S^3$ cells was determined.[11] The compounds were tested at 25, 50 and 100 μM for inhibition of DNA synthesis over 60 min [3]. The assay reaction was stopped with 1 N PCA + 1% Na P~P, collected on GF/A filters and counted. Select studies were conducted with compound **2** at 100 μM with and without VP-16 at 40 μM in an analogous manner.

### DNA Strand Scission

The effects of compounds **1-7** on DNA strand scission was determined by the methods of Suzuki *et al.*[4], Pera *et al.*[5] and Woynarowski *et al.*[6]. L1210 lymphoid leukemia or HeLa-$S^3$ uterine cells were incubated with 10 μCi thymidine methyl-$^3$H, 84.0 Ci/mmol for 24 hr at 37°C. L1210 cells ($10^7$) were harvested and then centrifuged at 600 g X 10 min in PBS. They were later washed and suspended in 1 ml of PBS. Lysis buffer (0.5 ml; 0.5 M NaOH, 0.02 M EDTA, 0.01% Triton X-100 and 2.5% sucrose) was layered onto a 5-20% alkaline-sucrose gradient (5 ml; 0.3 M NaOH, 0.7 KCl and 0.01 M EDTA); this was followed by 0.2 ml of the cell preparation. After the gradient was incubated for 2.5 hr at room temperature, it was centrifuged at 12,000 rpm at 20°C for 60 min (Beckman rotor SW60). Fractions (0.2 ml) were collected from the bottom of the gradient, neutralized with 0.2 ml of 0.3 N HCl, and radioactivity maesured. Thermal calf thymus DNA denaturation, U.V. absorption from 220 to 340 nm and DNA viscosity studies were conducted after incubation of compounds **1-7** at 100 μM at 37°C for 24 hr.

### P4-Phage Knotted DNA

P4-phage knotted DNA was isolated by the method of Liu and Davis [7]. Cultures of C-117 *E. coli* were grown overnight in 5 ml modified Luria Broth (1% bactotryptone, 0.5% yeast extract, 1% NaCl, 1.6 mM

$MgCl_2$, 0.5 mM $CaCl_2$, and 1% dextrose). After 18 hr, P4 phage 1:4000 stock dilution (4 ml, ~$1x10^{11}$ pfu/ml) was used to inoculate the 5 ml overnight culture. Phage was allowed to adsorb by sitting at room temperature for 10 min. Two L of modified Luria Broth was then inoculated with 4 ml phage infected overnight culture. The 2 L inoculate was incubated at 37°C with vigorous aeration for 4 hr and 20 min, after which 20 ml 0.5 M EGTA (pH 8) was added. After 1 hr of stirring at 37°C, chloroform (4 ml) was added to ensure complete cell lysis. The entire 2 L of inoculated broth was centrifuged, in 40 ml aliquots, at 10,000 rpm and 4°C, for 10 min, to remove cell debris. The final volume of supernatant collected was measured and sufficient NaCl, PEG-8000, and $MgCl_2$ were added to make a solution of 0.5 M NaCl, 10% PEG-8000, and 80 mM $MgCl_2$. This mixture was allowed to precipitate at 4°C overnight. After 18 hr the precipitate was pelleted by centrifugation at 10,000 rpm for 10 min. Pellets were combined and resuspended in 15 ml P4 diluent [ 1% $NH_4OAc$, 80 mM $MgCl_2$, 10 mM Tris-HCl (pH 7.2)] and repelleted. This process was repeated. Supernatants from the previous procedure were collected and centrifuged at 25,000 rpm for 4 hr. The resulting pellet, containing phage capsids and phage heads, was resuspended in 45 ml of P4 diluent and the phage solution was weighed. For every gram of phage solution, 0.632 g ultra pure CsCl was added. The CsCl/phage solution was placed in ultracentrifuge tubes and topped off with mineral oil. The CsCl/phage solution was centrifuged at 24,000 rpm, 4°C, for 3 days. Two "milky" colored bands were apparent after 3 days; upper band = P4 phage capsids, lower band = P4 heads (containing knotted DNA). The bands were collected via a syringe and were dialyzed with P4 diluent (2 L) using 18 mm dialysis tubing. P4 heads were extracted with an equal volume of ultrapure buffered phenol. The aqueous layer was separated and 2 volumes of 99 % ethanol were added and samples were placed at -20°C overnight to precipitate the knotted DNA. The precipitate was pelleted at 10,000 rpm for 5 min and was resuspended in 10 mM Tris (pH 8), 1mM EDTA, 0.1 M NaCl.

**DNA-Topoisomerase II**

L1210 or HeLa-$S^3$ DNA-topoisomerase II was isolated by the method of Miller, *et al.*[8] by pelleting approximately $10^8$ cells by centrifugation at 2500 rpm for 4 min. This pellet was then washed by resuspending in sterile PBS (pH 7.4) and pelleting again. Next, the pelleted cells were resuspended in buffer solution containing 0.25 M sucrose, 20 mM potassium phosphate (pH 7.5), 2 mM $MgCl_2$, 1 mM dithiothreitol (DTT), 1 mM spermidine, 0.1 mM EDTA, 0.1 mM phenylmethylsulfonyl fluoride (PMSF), and 1 mM $Na_2S_2O_5$, at 4°C. Cell membranes were lysed by addition of a volume of Triton X-100, equivalent to $1/100^{th}$ of the total cell suspension, in concert with Dounce homogenization. Complete cell lysis was determined by visualization of a small aliquot of cell homogenate stained with trypan blue (0.4%). An equal volume of buffer solution containing 1.75 M sucrose was added to cell suspension and mixed by gently swirling. After mixing the total volume was loaded on a sucrose cushion, 1.4 M sucrose, 20 mM potassium phosphate (pH 7.5), 5 mM $MgCl_2$, 1 mM DTT, 0.1 mM EDTA, 0.1 mM PMSF, and centrifuged at 18,000 rpm for 45 min, at 4°C. The sucrose cushion was removed via vacuum aspiration and the remaining pellet was resuspended in buffer containing 20 mM potassium phosphate (pH 7.5), 2 mM $MgCl_2$, 1 mM DTT, 0.1 mM EDTA, 0.1 mM PMSF, 1 mM β-mercaptoethanol, 10% glycerin, and 100 mM NaCl. The suspension was incubated at 4°C for 30 min and then was centrifuged at 2,400 rpm for 5 min. Supernatant was collected and the pellet was resuspended in buffer containing 150 mM NaCl and incubated again for 30 minutes at 4°C, followed by centrifugation. The process was repeated with buffer containing 400 mM NaCl. Fractions containing active enzyme, as determined by the topoisomerase unknotting assay, were kept and stored, after the addition of glycerin to make a 50% v/v solution, at -70°C. Active fractions were diluted to 1 U/μl (1 U was defined as the amount of enzyme required to completely unknot 150 ng knotted DNA in 1 hr at 37°C) with buffer solution.

**DNA Unknotting Assay**

The effects of the agents on isolated L1210 or HeLa-$S^3$ DNA topoisomerase II activity was determined by the method of Miller *et al.*[8]. The reaction mixture was prepared to contain the following components at the following concentrations: 0.05 M Tris (pH 7.5), 0.1 M KCl, 0.01 M $MgCl_2$, 30 μg/ml bovine serum albumin, 0.5 mM EDTA, 1.0 mM DTT, 1.0 mM ATP. A "4 x Reaction Mix" was prepared, meaning the components were four times as concentrated as the previously described mix. To prepare samples a premix was made containing 2.5 μl "4 x Reaction Mix" per sample, 0.25 μl knotted

DNA/sample, and enough autoclaved, distilled water to bring the volume of each sample up to 8.0 µl. To each sample reaction 1.0 µl sample compound (at desired concentration) and 1.0 µl topoisomerase II (1 U/µl) was added so that the final volume of each sample reaction was 10 µl. The samples were allowed to incubate at 37°C for 1 hr, at which time 2.5 µl/sample of a stop / loading buffer [50% w/v sucrose, 0.5% w/v sodium dodecylsulfate (SDS), and 0.25% w/v bromophenyl blue] was added to stop the reaction. Each sample, along with an enzyme and DNA control sample, was run for 18 hr using a 0.7% agarose gel, in electrophoresis buffer (pH = 8.0) [90 mM Tris, 2 mM EDTA, 90 mM boric acid], on a Gibco BRL Horizon 11 X 14 electrophoresis apparatus at 23 v. VP-16 (etoposide) was used as an internal standard for DNA topoisomerase II inhibitors. Photographs of gels were made by illumination of gels on a UV light table using Polaroid 667 film. Densitometric analysis was performed by using a GS 300 Transmittance / Reflectance Scanning Densitometer and the GS 365 Densitometry Program (version 2) for personal computers (Hoefer Scientific Instruments, San Francisco, CA). Photographs of agarose gels were scanned by the densitometer, in reflectance mode, perpendicularly to the direction of DNA migration, aligned with the unknotted DNA bands. To standardize the quantification of unknotted DNA, known amounts of completely unknotted P4 DNA were electrophoresed, photographed, and densitometrically scanned. The area under the curves corresponding to unknotted DNA bands were calculated using the densitometry software and were plotted versus nanograms of unknotted DNA. Amounts greater than 100 ng of unknotted DNA the unknotted DNA band intensity had plateaued and there was no further increase in intensity with increasing amounts of unknotted DNA.

**Protein-Linked DNA Break**

The production of protein-linked DNA breaks, by the agents, was evaluated by the method of Rowe *et al.*[9]. Approximately $10^7$ L1210 or HeLa-$S^3$ cells, growing in 50 ml RPMI-1640 with L-glutamine, 20% fetal calf serum, penicillin (100,000 U/ml) and streptomycin (100,000 g/ml), were inoculated with 50 µCi [6-$^3$H]-thymidine (15.0 Ci/mmol) and allowed to incubate 20-24 hr. The cell suspension was then centrifuged at 2,000 rpm for 10 min to pellet the cells. The supernatant was removed by vacuum aspiration and the cells were resuspended in fresh media, 37°C. The resuspended cells were allowed to incubate at 37°C for 2-3 hr to wash out any unincorporated [methyl-$^3$H]-thymidine. Then the cells were equally divided among sample tubes, 0.125 ml/sample, four tubes per controls or sample compounds. Controls include tubes, which contained no drugs, and tubes which contained VP-16 (final VP-16 concentration was 40 µM). Sample compounds (200 µM) in media, 0.125 ml, were added to 0.125 ml of the cell suspension, so that the final concentration was 100 µM. All samples were allowed to incubate 1 hr, at 37°C then 0.25 ml media containing drugs at 100µM were added. Incubation continued for 1 hr additionally. Then 0.5 ml doubly concentrated lysis buffer [2.5% SDS, 10 mM EDTA (pH 8), 0.8 mg/ml calf thymus DNA] was added. Samples were warmed to 65°C in a water bath and each was syringed eight times, to sheer DNA fragments to uniform size, using a 3 ml syringe equipped with a 22 gauge needle. Aliquots, 10 µl, were taken from each control tube containing no drugs, and were precipitated on glass-fiber filters (GF/A) with ice cold 5% TCA, to determine the total number of dpm per sample. To precipitate SDS, 0.25 ml 325 mM KCl was added and mixed by inversion. The samples were placed on ice for 10 min. Samples were centrifuged for 1 min on a Fisher Scientific micro centrifuge (model 235C) to pellet the precipitate. The supernatant was removed via vacuum aspiration followed by addition of a wash buffer [10 mM Tris (pH 7.5), 2 mM EDTA (pH 8), 100 mM KCl, 0.1 mg/ml calf thymus DNA]. The precipitate was resuspended by vortexing and the samples were placed in a 65°C water bath for 10 min. After the precipitate was completely dissolved, samples were placed on ice. After 5 min samples were vortexed, and returned to ice for another 5 min. Microcentrifugation for another minute was followed by removal of supernatant. The remaining pellets were then dissolved in distilled water at 65°C and transferred to scintillation vials. Scintiverse BD®, 4 ml, was added to each scintillation vial and samples were counted on a Packard Tricarb 4000 liquid scintillation spectrometer, and corrected for quenching.

**Interference Assay**

The interference assay followed the procedures described for protein-linked DNA breaks, only differing in that after the 1 hr incubation with sample compound, an equal volume of VP-16 (80 µM) plus an equimolar concentration of the appropriate sample compound was added to each sample [9]. The cells were allowed to incubate at 37°C for an additional hr after the addition of VP-16 solution. The cells were then lysed and processed exactly as described above.

**Cleavage-interference** *in vitro*

The method used to assess interference with drug-induced topoisomerase II-mediated DNA cleavage in vitro, was based on the procedure of Harker, et al.[10]. The Hind III-cut PBR322 DNA was end-labeled with [$\alpha$-$^{32}$P] dCTP (3000 Ci/mmol, ICN) using a commercial T4 polymerase labeling system. Cleavage reactions of 50 ml contained 20 ng/ml labeled DNA, 30 mM Tris-HCl [pH 7.6], 60 mM KCl, 8mM $MgCl_2$, 3 mM ATP, 15 mM β-mercaptoethanol, and 30 mg/ml nuclease-free BSA. Topoisomerase II (4 U) was added to the enzyme control reaction and drug treatments, the latter being 40 mM VP-16 in the absence or presence of amine borane derivatives. After a 30 min incubation at 37°C, reactions were divided equally and prepared for gel analysis with autoradiography and for quantifying protein-linked DNA breaks using $K^+$/SDS precipitation.

**Dilute agar colony method**

Following the modified method of Chu and Fischer [11], murine L1210 lymphoid leukemia cells were grown suspended in agar as colonies. Cells (1 x $10^5$), in RPMI-1640 with 10% fetal bovine serum, and penicillin / streptomycin / amphotericin B (2 ml), were incubated for 1 hr at 37°C in the presence of varying concentrations of experimental drugs, for drug treatments, or drug vehicle, DMSO, for controls. Following incubation, cells were pelleted by centrifugation at 600 x g for 5 min. The media was removed via aspiration, and the cell pellet was resuspended in fresh media of a volume equivalent to the original (2 ml). The cells were diluted by serial dilution to a concentration of 1 x $10^3$ per milliliter. The final dilution being made in sterile test tubes with a 0.13% agar solution of growth media, heated to 45°C. Agar dilutions were mixed by inversion and placed in test tube racks for incubation at 37°C. Following 5 days of incubation, a solution of 0.01% neutral red (200µl) in growth media was gently layered on the surface of each sample to diffuse into the colonies. On day 7, samples were decanted into 6-well plates and colonies were counted using a colony counter over a 2 mm x 2mm grid. Results were expressed as the percent of the number of colonies in untreated control.

**Phosphorylation of Topoisomerase II**

Phosphorylation of L1210 DNA or human topoisomerase II enzyme was measured by the method of DeVore *et al.*[12]. Reactions containing reaction buffer (100µg/ml phosphatidyl serine, 50 mM Tris HCl (pH 8.0), 25 mM NaCl, 7 mM $MgCl_2$, 100µM $CaCl_2$, and 30µM ATP, rat brain protein kinase C (4.55 U) (Sigma/Aldrich Chemical Co., St. Louis, MO), L1210 Topoisomerase II (4U/reaction), 10 µCi [$\gamma$-$^{32}$P]-ATP (3000 Ci/mmol) (Dupont / New England Nuclear, Reston, VA) and experimental drugs were allowed to incubate for 30 min or 2 hr at 37°C. Reactions were terminated by the addition of 1 ml 10% TCA. The acid insoluble precipitate was filtered onto GF/A glass fiber filter disks and counted for radioactivity. Results were expressed as percent of dpm of the untreated control. Standard protein kinase inhibitors A3 and bisindolylmaleimide (Calbiochem-Novabiochem International, San Diego, CA) were also assayed.

**VP-16 Uptake and Efflux from L1210 Cells**

[$^3$H (G)]-VP-16 [1mCi] [etoposide, 300-900 mCi, Morevek Biochemicals] was incubated with 2.5 x $10^6$ cells for 5 to 24 hr in RPMI-1640 + 15% FCS + P/S with compound **2** at 100 µM. Aliquots were removed at time intervals from the reaction mixture, washed and counted according to the method of Feng et al. [13]

**Statistics**

The mean and standard deviation are designated by "X ± SD." The probable level of significance (p) between test and control samples was determined by the Student's "t" test with the raw data.

### Results

Piperidine carboxyborane **1**, piperazine carboxyborane **2**, morpholine carboxyborane **3**, N-methylmorpholine carboxyborane **4**, 4-phenyl piperidine carboxyborane **5**, 4-methyl piperidine carboxyborane **6**, and N-methyl imidazole carboxyborane **7** were effective cytotoxic agents in the L1210 and HeLa-$S^3$ tumor screens. All of the $ED_{50}$ values were less than 4 μg/ml with the exception of compound **3** in the L1210 screen [Table 2]. Generally the compounds were more effective in the HeLa-$S^3$ uterine carcinoma screen and were very comparable with standard antineoplastic agents used in these screens.

**Table 2 Cytotoxic Evaluation of Heterocyclic Amine Carboxyboranes**

| | N = 4 | $ED_{50}$ = μg/ml |
|---|---|---|
| Compound | L1210 | HeLa-$S^3$ |
| 1 | 1.74 | 1.92 |
| 2 | 3.34 | 1.60 |
| 3 | 4.44 | 2.14 |
| 4 | 2.68 | 2.48 |
| 5 | 3.56 | 1.23 |
| 6 | 2.85 | 1.71 |
| 7 | 2.46 | 1.97 |
| 5FU | 1.41 | 2.47 |
| Ara-C | 2.76 | 2.13 |
| Cycloleucine | 2.67 | 1.98 |
| Hydroxyurea | 2.67 | 1.96 |

**Fig. 1**

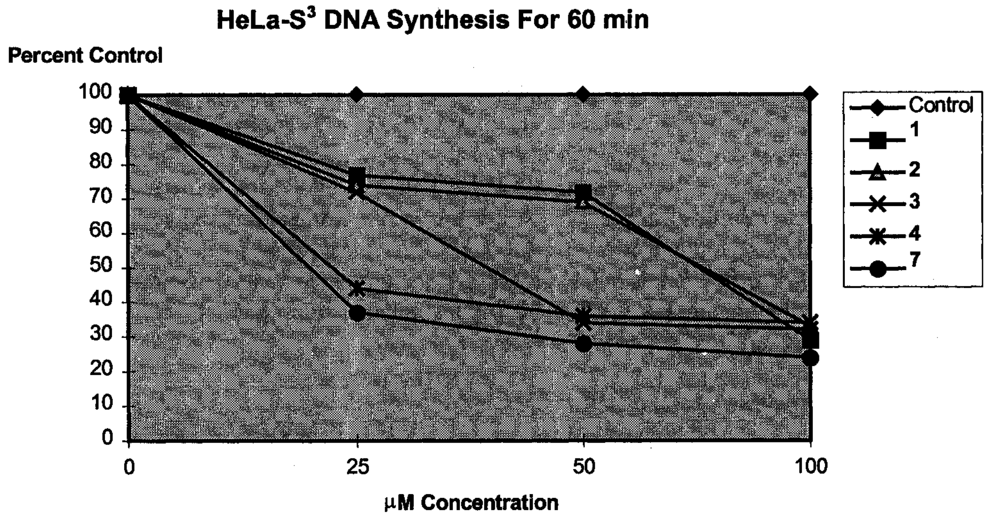

The compounds were effective in reducing DNA synthesis greater than 50% in both the HeLa-$S^3$ uterine carcinoma and L1210 lymphoid leukemia cells over 60 min [Fig. 1 and 2].

Fig. 2

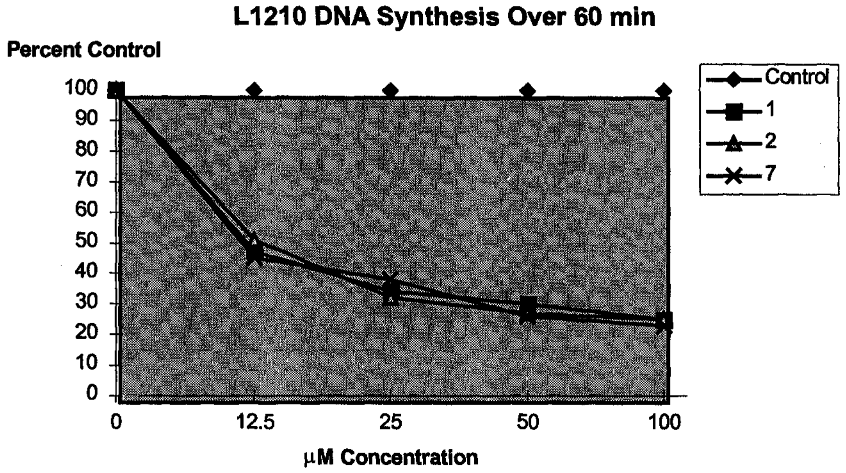

Fig. 3

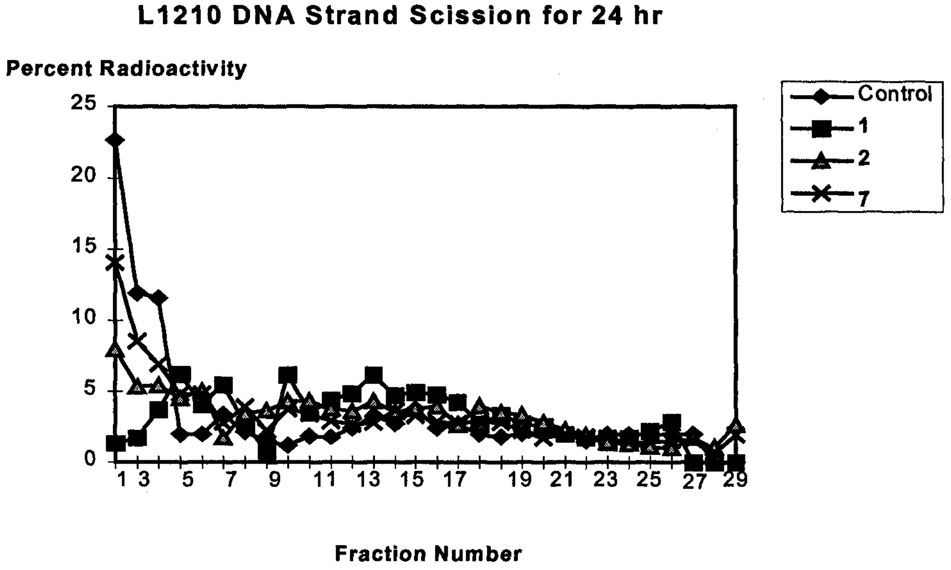

DNA strand scission was evident in both cell lines after 24 hr. incubation at 100 μM with smaller fragments appearing in the gradient of both L1210 and HeLa-$S^3$ cells [Fig. 3 and 4].

Using the partially purified HeLa-$S^3$ DNA topoisomerase II enzyme, subsequent studies demonstrated that the compounds **1-7** were inhibitors at ~200 μM. [Fig 5]. Using the L1210 partially purified DNA topoisomerase enzyme was inhibited at $\geq$ 100 μM for the compounds. An in-depth study was performed to determine if the compounds affected DNA topoisomerase II activity in a concentration range consistent with the concentration needed for DNA synthesis inhibition. Compound **2** was selected for this detailed study.

**Fig. 4**

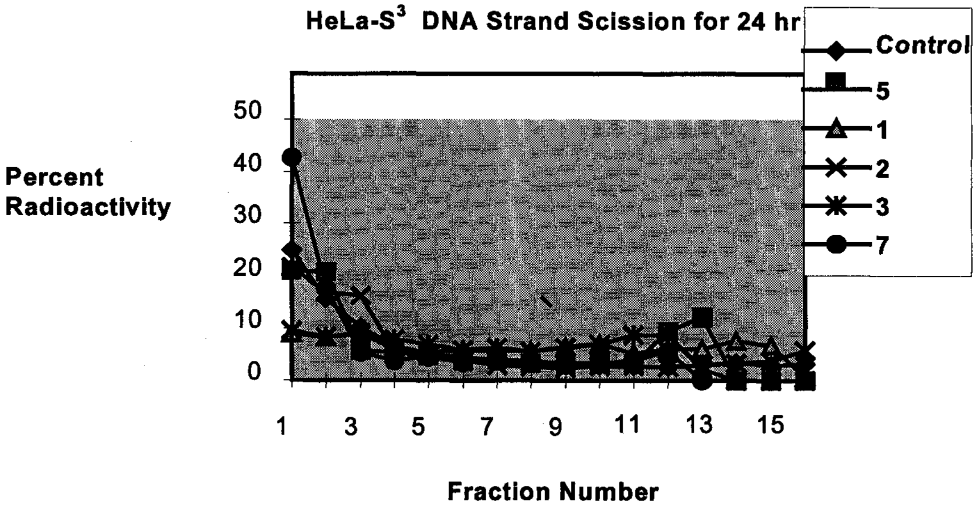

**Fig. 5 HeLa-S³ DNA Topoisomerase II Inhibition**

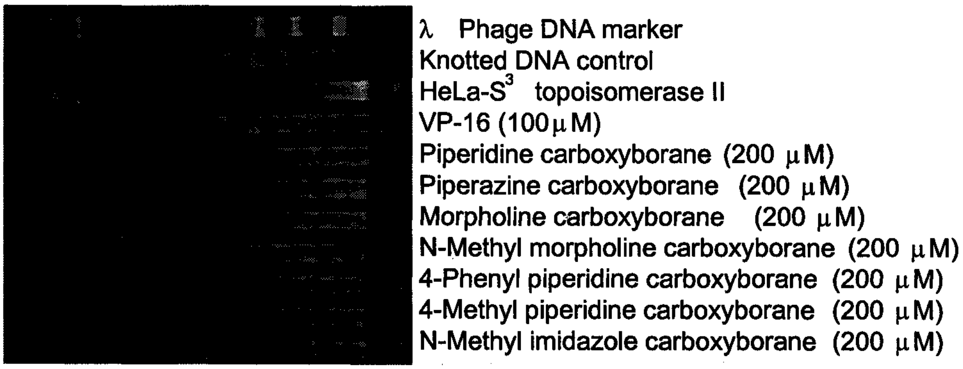

**Fig. 6**

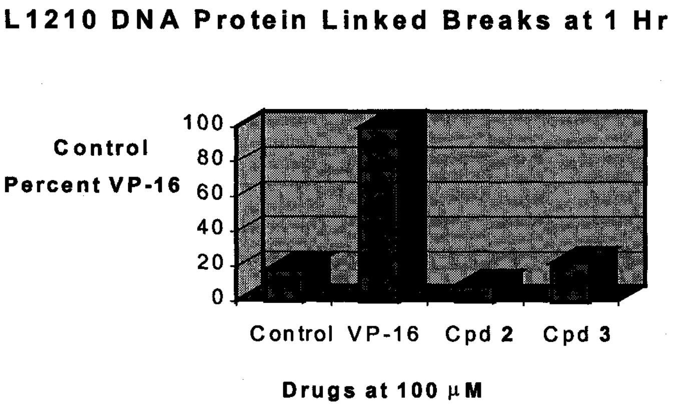

**Fig. 7**

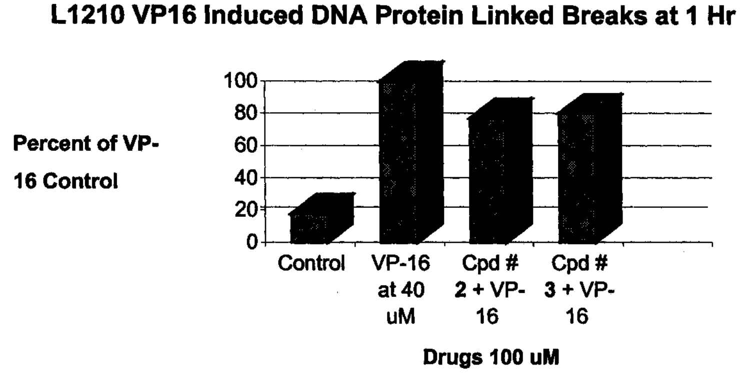

L1210 cell DNA protein linked breaks were not evident after 1 hr incubation with compounds **2** or **3** at 100 μM, alone [Fig 6], but the standard VP-16 at 40 μM did afford breaks at 1 hr. Combinations of VP-16 (40 μM) with compounds **2** or **3** (100 μM) did not demonstrate an increasing number of DNA linked breaks through 3 hr [Fig 7]. On the other hand, compounds **2** and **3** appeared synergistic with VP-16 affording additional breaks at 15 and 24 hr which were significantly greater in magnitude than with VP-16 alone [Fig. 8 and 9]. These studies suggested that the heterocyclic amine carboxyboranes functioned

**Fig. 8**

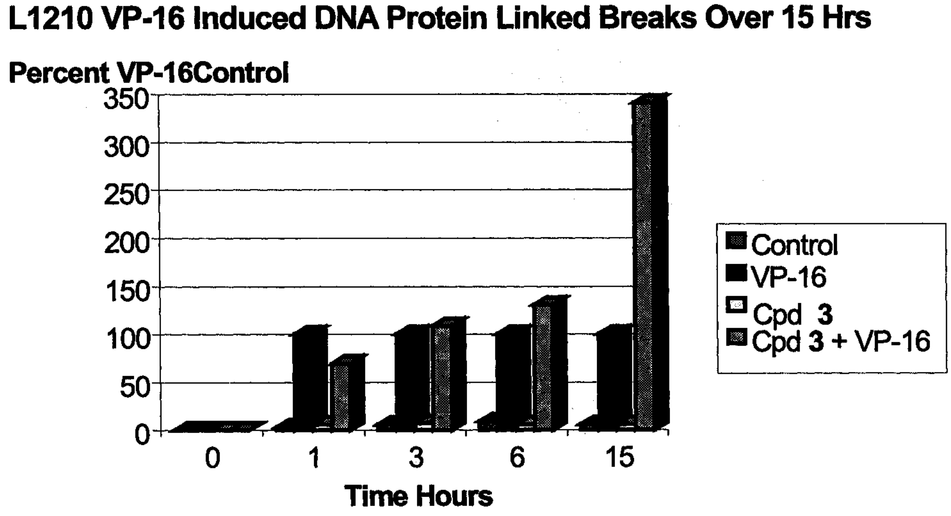

in a manner different than VP-16 in affording DNA protein linked breaks and this effect was delayed after the observed DNA synthesis inhibition at 1 hr of ~ 50%.

The Hind III-cut PBR322 DNA end-labeled with [α-$^{32}$P] dCTP study also confirmed that compound **2** was not a direct inhibitor of DNA topoisomerase II activity from 1 to 100 μM, but it does appear to be enhancing the action of VP-16 causing DNA breaks at 10 and 100 μM suggesting that the heterocyclic amines function by some other mechanism than binding to the same site on the enzyme as VP-16 [Fig 10].

**Fig. 9**

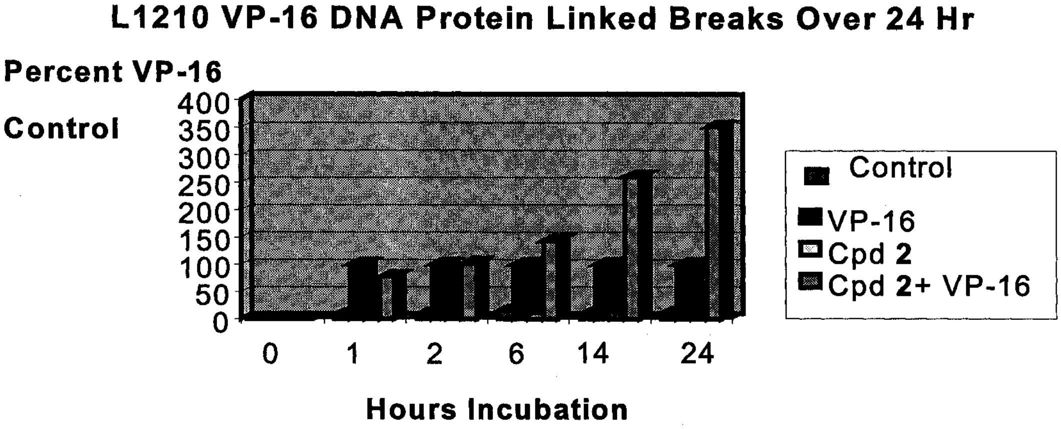

On the other hand, combinations of VP-16 at 40 μM and compound **2** at 100 μM were shown to enhance L1210 cell death [Fig 11], inhibit L1210 DNA synthesis [Fig 12] and reduce dilute agar colony growth of surviving cells [Fig 13]. This enhancement of the reduction of these cellular

**Fig. 10**

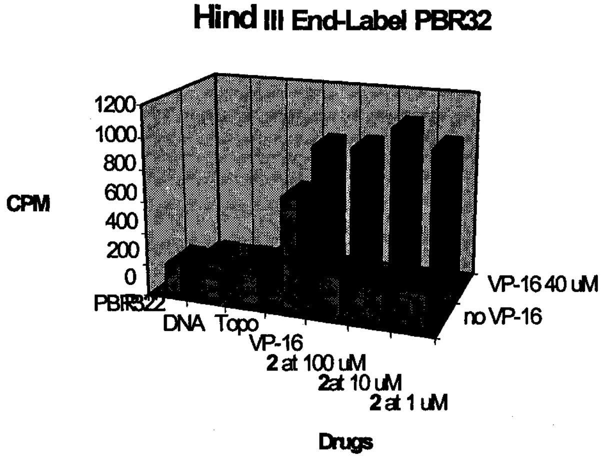

parameters was not equal to a simple addition of the effects of VP-16 + the agent. By using $^3$H VP-16, the uptake and efflux of VP-16 in L1210 cells was shown not to be enhanced by the presence of compound **2** at 100 μM [Fig 14 ]. In fact, compound **2** reduced the concentration of labeled VP-16 uptake into the L1210 cells after 6 min. Thus, the compound did not cause the accumulation of VP-16 in the cancer cell thus causing more cytotoxic effects by this mechanism.

Combination studies of VP-16 with compound **2** demonstrate that use of both agents together produce a significant increases in small fragments of DNA in the gradient [Fig 15]. VP-16 alone caused DNA fragmentation with medium size DNA fragments, whereas when the drug was added in combination this resulted in smaller DNA fragments. Nevertheless, the drug alone was capable of producing small fragments of DNA. The phosphorylation by protein kinase C of L1210 DNA topoisomerase II as well as human DNA topoisomerase II was found to be reduced by compound **2** whereas VP-16 caused an elevation of the phosphorylation by PKC. The magnitude of reduction afforded by compound **2** was similar to known inhibitors of protein kinase C activity [Table 3] and was delayed in time with the highest inhibition of phosphorylation by PKC at 2 hr.

**Fig. 11** **L1210 Cell Growth**

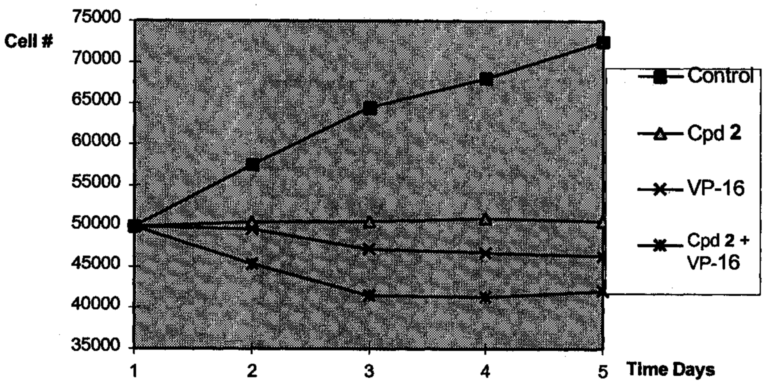

**Fig. 12**

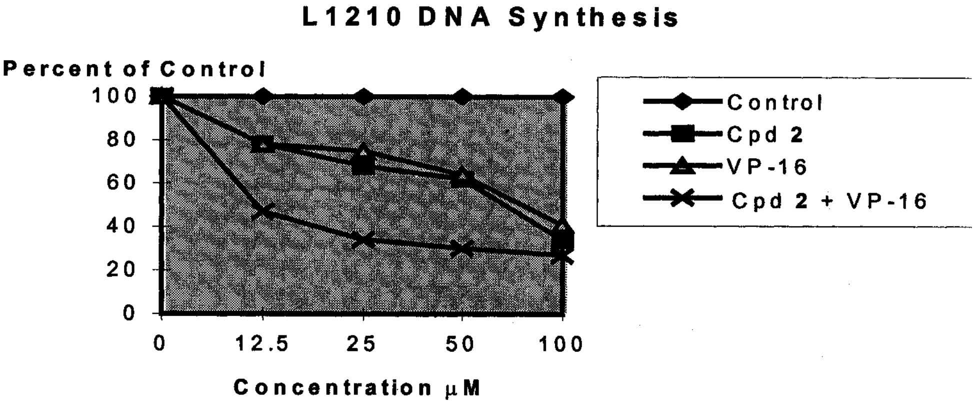

**Fig. 13**

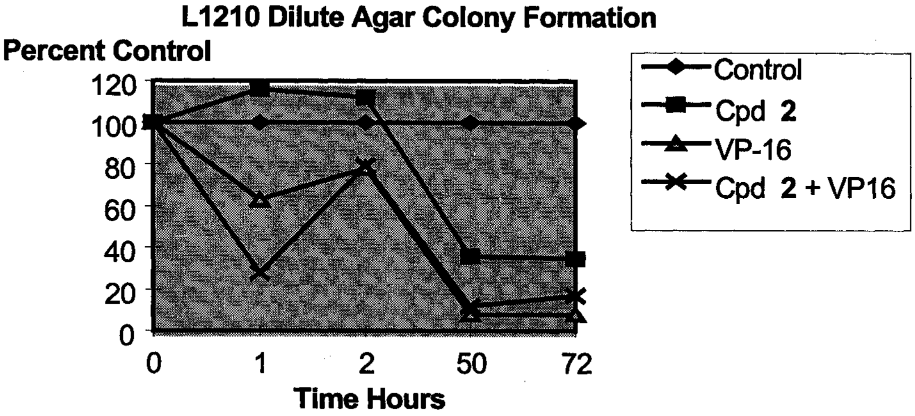

**Fig. 14**

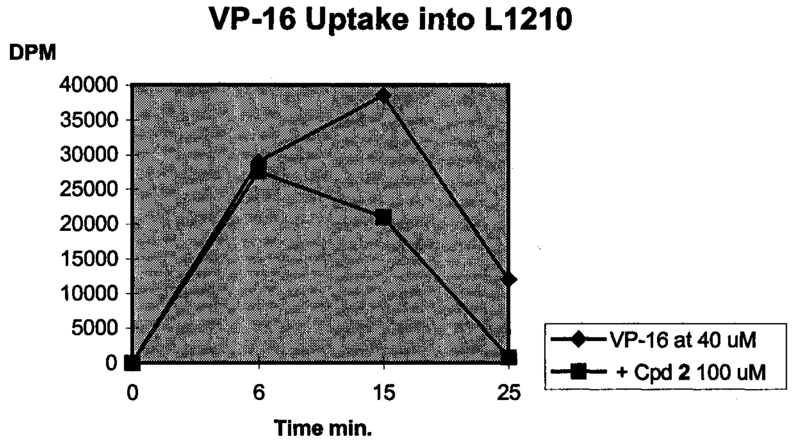

**Fig. 15**

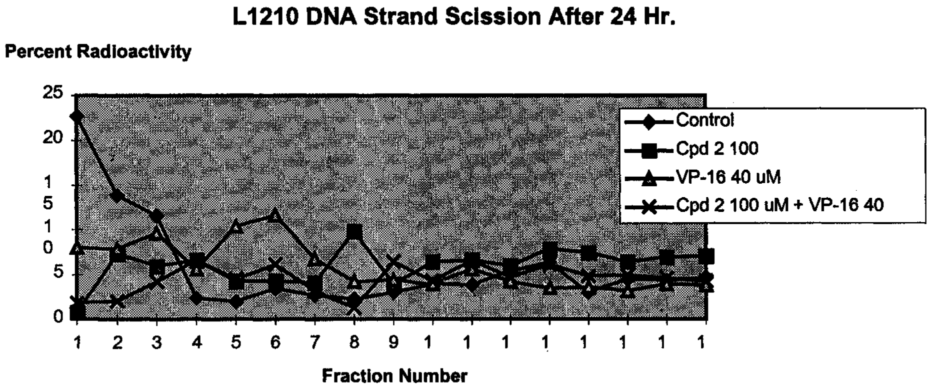

**Table 3: Inhibition of Protein Kinase C-Dependent Phosphorylation of $L_{1210}$ and Human DNA Topoisomerase II by Amine Carboxyborane Derivatives After a 0.5 or 2 Hr Exposure.**

| | $L_{1210}$ Topoisomerase II | | Human Topoisomerase II | |
|---|---|---|---|---|
| | 30 min mean ± s.d. | 2 hr mean ± s.d. | 30 min mean ± s.d. | 2 hr mean ± s.d. |
| Control | 100 ± 16[a] | 100 ± 8[b] | 100 ± 23[c] | 100 ± 17[d] |
| 2 (100 μM) | 141 ± 21 | 78 ± 9 * | 79 ± 13 | 70 ± 16 |
| VP-16 (100 μM) | 157 ± 40* | 160±70* | -- | -- |
| Bisindolylmaleimide I (20 nM) | 27±3* | 18 ±3 *** | 12 ± 1 ** | 4 ± 1 *** |
| A3 (100 μM) | 77 ± 3* | 66 ± 14 * | 36 ± 4 ** | 68 ± 14* |

(N = 3); * $p < 0.05$; ** $p < 0.02$; *** $p < 0.001$; [a] 1590 cpm; [b] 418 cpm; [c] 1847 cpm; [d] 14050 cpm

**Discussion**

The heterocyclic amine carboxyborane adducts have more than one mode of action in affording tumor cell death. Obviously the reported inhibition of enzyme activities involved in the regulation of the purine pathway by the compounds from 25 to 100 μM in L1210 cells will result in cell death by reducing deoxyribonucleotide pools [1]. DNA strand scission has also been linked to tumor cell death or apoptosis. Since the L1210 growth study demonstrated that longer than 24 hr was required to observe significant changes in cell number in the presence of these compounds, this suggests that the heterocyclic amine carboxyboranes are altering cellular biochemical events later than 60 min. Thus, there are secondary events afforded by the drugs which are also important in causing cancer cell death. These agents do not appear to be L1210 DNA topoisomerase II inhibitors at 100 μM concentrations consistent with the concentration of drug for the observed inhibition of DNA synthesis and DNA fragmentation. The agents were additive with the effects of VP-16 in inducing DNA protein linked breaks after 6 hr incubation in a concentration dependent manner. The study would suggest that the heterocyclic amine carboxyboranes did not bind to the same site on DNA topoisomerase II enzyme as VP-16 since they produced no cleavage product when used alone from 1 to 24 hr. The agents appeared to be acting in a manner other than VP-16 on the DNA topoisomerase II enzyme to enhance the ability of VP-16 to afford cleavable product, i.e. DNA protein linked breaks. VP-16 is passively taken up by cancer cells but its efflux is by the p-glycoprotein transporter. This transporter can be blocked by a variety of agents, i.e. verapamil, to block the efflux of VP-16 and increase cell death. However, the heterocyclic amine carboxyboranes did not cause increased accumulation of VP-16 in L1210 cells. Agents which block phosphorylation by PKC of DNA topoisomerase II reduce its catalytic action and cause more DNA protein linked breaks followed by apoptosis [12, 14-15]. These studies have demonstrated that VP-16 actually stimulates phosphorylation of L1210 and human DNA topoisomerase II whereas the heterocyclic amine carboxyboranes cause a reduction in phosphorylation of the enzyme which is related to increase in cleavable products and cell death.

**Acknowledgment**

The authors wish to thank the Elsa Pardee Foundation and the North Carolina Biotechnology Institute for supporting this research.

### References

1. C.K. Sood, A. Sood, B.F. Spievogel, J.A. Yousef, B.S. Burnham, and I.H. Hall, *J. Pharm. Sci.* **80**, 1133, (1991).
2. R.J. Geran, N.H. Greenburg, M.M. MacDonald, A.M. Schumacher and B.J. Abbott, *Cancer Chemo. Rep.* **3**, 9, (1972).
3. L.L. Liao, S.M. Kupchan, S.B. Horwitz, *Mol. Pharmacol.* **12**, 167, (1976).
4. H. Suzuki, T. Nishimura, S.K. Muto and N. Tanaka, *J. Antibacteriol.* **32**, 875 (1978).
5. J.F. Pera, Sr, C.J. Rawlings, J. Shackleton and J.J. Roberts, *Biochem. Biophy. Acta* **655**, 152 (1981).
6. J.W. Woynarowski, T.A. Beerman and J. Konopa, *Biochem. Pharmacol.* **30**, 3005 (1981).
7. L.F. Liu, T.C. Rowe, L. Yang, K.M. Tewey and G.L.Chen, *J. Biol. Chem.* **258**, 15365 (1983).
8. G. Miller, L.F. Liu, and P.T. England, *J. Biol. Chem.* **256**, 9334-9339, (1981).
9. T.C. Rowe, G.L. Chen, Y.H. Hsiang and L.F. Liu, *Cancer Res.* **46**, 2021 (1986).
10. W.G. Harker, D.L. Slade, F.H. Drake, and R.L. Parr, *Biochemistry* **30**, 9953, 1991.
11. M. Chu and G.A. Fischer, *Biochem. Pharm.* **17**, 753-767, (1968).
12. R.F. DeVore, A.H. Corbett and N. Osheroff, *Cancer Res.* **52**, 2156, (1992).
13. M.R. Feng, M. Liebert, G. Wedermeyer, H.B. Grossman, W.R. Manicini, M. Williams, J.G. Wager, *Selective Cancer Therapeutics* **7**, 75, 1991.
14. P. Ackerman, C.V. C. Glover, and N. Osheroff, *Proc. National Acad Sci.* **82**, 3164(1985).
15. R.Bertrand, D. Kerrigan, M. Srang, and Y. Pommier, *Biochem. Pharmacol.* **42**, 77,(1991).

**Received: May 15, 1997 - Accepted: May 28, 1997 - Received in revised camera-ready format: June 18, 1997**

